# Modification of Residual Stresses in Laser Additive Manufactured AlSi10Mg Specimens Using an Ultrasonic Peening Technique

**DOI:** 10.3390/ma12030455

**Published:** 2019-02-01

**Authors:** Xiaodong Xing, Xiaoming Duan, Xiaojing Sun, Haijun Gong, Liquan Wang, Fengchun Jiang

**Affiliations:** 1 College of Mechanical and Electrical Engineering, Harbin Engineering University, Harbin 150001, China; wangliquan@hrbeu.edu.cn; 2 College of Materials Science and Chemical Engineering, Harbin Engineering University, Harbin 150001, China; sunxiaojing@hrbeu.edu.cn (X.S.); jiangfengchun@hrbeu.edu.cn (F.J.); 3 Manufacturing Engineering Department, Georgia Southern University, Statesboro, GA 30460, USA; hgong@georgiasouthern.edu

**Keywords:** selective laser melting, additive manufacturing, ultrasonic peening treatment, residual stress, numerical simulation

## Abstract

Ultrasonic peening treatment (UPT) has been proved to be an effective way of improving residual stresses distribution in weld structures. Thus, it shows a great potential in stress modification for metal parts fabricated by additive manufacturing technology. In this paper, an investigation into the ultrasonic treatment process of AlSi10Mg specimens fabricated by selective laser melting (SLM) process was conducted by means of experimental and numerical simulation. The specimens were prepared using a SLM machine, and UPT on their top surface was carried out. The residual stresses were measured with an X-ray stress diffraction device before and after UPT. Meanwhile, a finite element simulation method for analyzing the influence of UPT on the residual stress field of specimens was proposed and validated by experiments. Firstly, the thermal mechanical coupling numerical simulation of the SLM process of the specimen was carried out in order to obtain the residual stress distribution in the as-fabricated specimen. Then, the transient dynamic finite element simulation model of the UPT process of the specimen was established, and the UPT effect analysis was implemented. In the UPT simulation, the residual stress was applied as a pre-stress on the specimen, and the specimen’s material mechanical property was described by the Johnson–Cook model, whose parameters were determined by Split Hopkinson Pressure Bar (SHPB) experiment. The residual stress distribution before and after UPT predicted by the finite element model agree well with the measurement results. This paper concludes with a discussion of the effects of ultrasonic peening time, as well as the frequency and amplitude of the peening needle on residual stress.

## 1. Introduction

Metal additive manufacturing technology has been widely used since it has been introduced to the market, particularly in aerospace and biomedical applications. However, the residual stresses generated in continuous transient melting and solidifying process are still a significant barrier for additive manufacturing of high-performance large-size metal parts, which can lead to defects such as excessive deformation and cracking [1,2,3,4,5]. Therefore, the stress-relieving operations during or after the part construction process is necessary in the metal additive manufacturing. The Ultrasonic peening treatment (UPT) technology is used to modify the residual stress by applying high-frequency mechanical peening or vibration on the part surface, which can lead to plastic deformation near the surface [6]. In the early 1970s, the ultrasonic peening method was proposed by Statnikov [7] and it has been widely used in industrial fields such as aerospace, ship and ocean engineering, bridge structures, etc. [8,9,10,11] The International Welding Association has been showing great enthusiasm for the application of UPT to post-weld treatments to modify residual stresses [12]. UPT of the weld toe can improve the microstructure and optimize the geometry of the welding joint, so that tensile stresses in the surface areas can be transformed into compressive stresses after UPT, thereby the mechanical properties of parts such as hardness, fatigue life and strength can be improve greatly [13,14,15]. On account of the similarity of welding process and metal additive manufacturing technology, UPT is also very promising in the field of metal parts additive manufacturing [16,17].

Common methods for residual stress measurement include X-ray diffraction, contour method, incremental deep-hole drilling and ring-core technology [18,19,20,21]. By means of these measuring methods, a large number of experimental studies have been conducted on the effects of UPT on the residual stress field of the welded parts. Liu et al. [22] measured the interior and surface residual stress distribution of the welded specimen after UPT by means of contour method and X-ray diffraction, respectively. The results show that the compressive stresses generated by UPT in the welded area has the same effect in the lateral and longitudinal directions of the specimen, and the residual stress distribution is more uniform after the peening. Ficquet et al. [23] used the incremental deep-hole drilling method and the ring-core techniques to measure the residual stress distribution of the 50D steel specimens after the ultrasonic peening, respectively, at a depth of 0.5 mm, 1 mm and 2 mm. The experimental results indicate that the UPT technology can generate a zone near the surface and the maximum depth up to 2 mm, where the tensile stresses are transformed to the compressive stresses. Yu et al. [24] performed UPT and corrosion tests on welded joints of 6005A-T6 aluminum alloy material. Ganiev et al. [25] used metallography methods to investigate the surface hardening and internal residual stress of specimens treated by ultrasonic peening. It was found that the formation of favorable compressive stress was accompanied by the fine crushing of crystalline grains in the narrow surface layer of the treated metal.

Because the residual stress measurement experiments are time consuming, costly and highly dependent on the operator’s skills, some researchers have carried out numerical simulation studies on the UPT process and residual stresses prediction. Yin et al. [26] used the finite element software ABAQUS to build the impact-rebound-impact model to predict the residual stress distribution of the specimen after ultrasonic peening. An elastoplastic model was used to describe the constitutive relationship of the material during UPT. The simulation results are basically consistent with the residual stress X-ray diffraction measurements in the experiment. Yuan and Sumi [27] used SYSWELD and LS-DYNA to implement the thermomechanical welding and elastoplastic ultrasonic peening simulation successively, and the welding residual stress field was treated as prestress field in the peening simulation. Khurshid et al. [28] studied the effects of UPT on residual stresses in S355, S700MC, and S960 grades steel experimentally and numerically based on different material models.

The aim of this article is to investigate residual stress modification for laser additively manufactured parts by UPT process. A numerical simulation method was developed to analyze the influence of UPT on the residual stress of specimens fabricated by SLM, including three-dimensional thermomechanical coupling SLM process simulation and transient dynamic UPT process of the as-fabricated specimens. The Johnson–Cook constitutive model was used in the ultrasonic peening process. The model can consider various complex factors such as ultrasonic peening process parameters and mechanical properties changes. In addition, a series of experimental studies were carried out to determine the parameters of the material model and measure the residual stress in order to validate and increase the reliability of the numerical simulation analysis strategy.

## 2. Ultrasonic Peening Treatment

### 2.1. The Basic Principle of UPT

**Figure 1:**
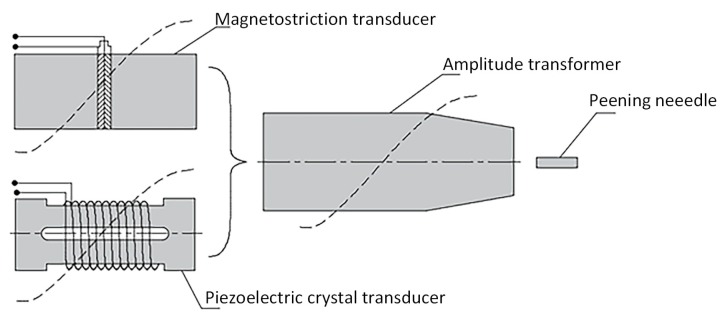
The schematic of principle of Ultrasonic peening treatment (UPT).

The basic principle of UPT technology is shown in Figure 1. The magnetostrictive or piezoelectric crystal transducer converts the high frequency alternating electrical energy into mechanical energy, and the vibration output end of the transducer is connected to the amplitude transformer, and the other end of the amplitude transformer has a certain gap with the peening needle. When the high-frequency alternating electric energy is input, the transducer performs a small amplitude longitudinal reciprocating motion, and the amplitude transformer expands the amplitude to drive the peening needle, and the peening needle starts to rebound after hitting the surface of the part. The peening needle thus reciprocates at a high speed and frequency between the amplitude transformer and the part, thereby completing the UPT of the surface of the part [6].

### 2.2. Johnson–Cook Model

As early as the 1950s, experts and scholars had carried out research on the application of ultrasonic shock to metal surface treatment [29,30]. In 1983, Johnson and Cook proposed a classic Johnson–Cook constitutive model when studying the problems of impact and penetration [31]. The model can describe the effects of strain, strain rate, and temperature on the yield stress and failure strain of the material during deformation. The high-speed nonlinear collision process between the peening needle and the parts leads to the work hardening, strain rate strengthening effect and temperature softening effect, which have significant influence on the material properties of the part. Therefore, the Johnson–Cook constitutive model in the software MSC Marc was adopted to consider the influence of the above factors on the material properties.

The Johnson–Cook model describes the material’s stress–strain relationship as follows:

(1) $$\sigma =\left(A+B\varepsilon^{n}\right)[1+C\ln(\dot{\varepsilon }/{\dot{\varepsilon }}_{0})]\left[1-{\left(\frac{T-T_{r}}{T_{m}-T_{r}}\right)}^{m}\right]$$

σ—stress, ε—plastic strain, $\dot{\varepsilon }$—strain rate, ${\dot{\varepsilon }}_{0}$—reference strain rate, *T*—temperature, *T_r_*—reference temperature 25 °C, *T_m_*—material’s melting point; *A*, *B*, *n*, *C* and *m* represent initial yield strength, strain hardening index, strain rate sensitivity coefficient, hardening index, and temperature softening index, respectively.

## 3. Experimental Study

### 3.1. Experiment Setup

**Figure 2:**
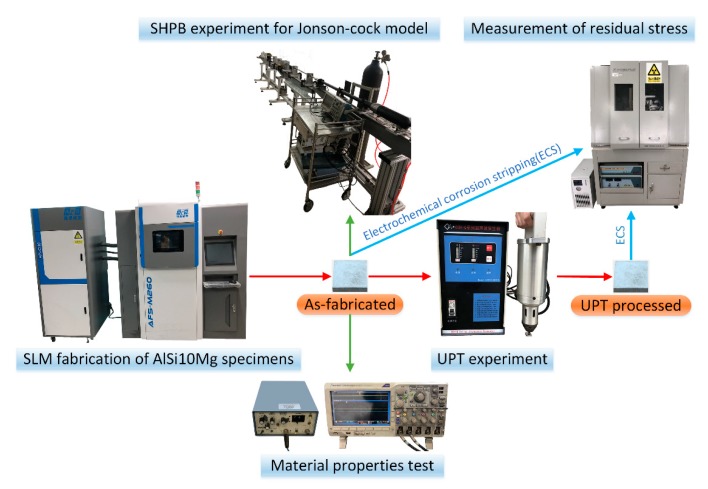
The schematic of experiment setup.

The preparation of specimens, UPT process and measurement of material properties and residual stresses were carried out. Accordingly, as shown in Figure 2, the experimental system consists of a SLM machine, a UPT device, a device of ultrasonic pulse transmitting and receiving, a digital oscilloscope, a SHPB device and an X-ray stress diffraction tester.

### 3.2. Specimens Building with SLM Process

AlSi10Mg specimens were fabricated by AFS-M260 SLM machine (from Beijing Longyuan Automatic Forming System Co., Ltd., Beijing, China). The specimens’ size is 12 × 12 × 4.5 mm, and the chemical compositions of aluminum powder (AlSi10Mg) was listed in Table 1. First, the STL file of the specimen model was sliced and input into the machine. Then the substrate was preheated to 120 °C, and the oxygen concentration in the building chamber was reduced to an acceptable value. The processing parameters are shown in Table 2.

### 3.3. UPT Process

The as-fabricated specimens’ surface was treated by high-frequency mechanical peening. The UPT device includes a GCH-Q ultrasonic generator (from Weihai Guosheng Ultrasonic Technology Co., Ltd., Weihai, China), and its working principle is shown as Figure 1. During the peening process, the peening head went through the specimen’s entire top surface manually, with its axis perpendicular to the surface. Excessive peening time, amplitude and frequency may lead to cracks produced inside the specimens. An insufficient amount of time, small amplitude or low frequency may reduce the effect of UPT. As shown in Table 3, the parameters used in UPT process are tailored to the mechanical properties of AlSi10Mg specimens empirically.

### 3.4. Measurement of Residual Stresses

The residual stress values of the specimens before and after UPT were measured by XRD equipment, X-350A residual stress tester (from Beijing Changliu Scientific Instrument Co., Ltd., Beijing, China). The residual stress measurement experiment was based on British standard BS EN 15305: 2008. In the measurement process, the surface of the sample was subjected to a stripping operation by electrolytic polishing to measure the surface stress at different depth in the thickness direction of the specimens. The polishing process was based on ASTM standard: E1558-09. After each stripping process, the center point of the sample surface was taken to measure the value of residual stress and used to validate the results in the simulations at the same location.

### 3.5. Measurement of Material Properties of the Specimen

The elastic modulus and Poisson’s ratio of the specimen were measured using an ultrasonic detector. The 12 × 12 × 4.5 mm specimens were prepared by SLM process, and the upper and lower surfaces were polished. Two probes of the detector were successively placed on the upper surface of the specimen, and the propagation time of transverse and longitudinal wave in the specimen along the thickness direction were obtained respectively. Then, using the formula *v* = 2*d t*^−1^, the wave velocities can be calculated. Item *d* is the thickness of the test piece. The density of the sample can be measured as well. Finally, the elastic modulus and Poisson’s ratio of the sample can be calculated by taking the measured data into Equations (2) and (3). The measured material property parameters are shown in Table 4.

(2) $$\nu =\frac{1-2{\left(V_{T}-V_{L}\right)}^{2}}{2-2{\left(V_{T}-V_{L}\right)}^{2}}$$

(3) $$E=\frac{{V_{L}}^{2}\rho \left(1+\nu \right)\left(1-2\nu \right)}{1-\nu }$$

where $V_{T}$, $V_{L}$, ρ, ν, E are transverse wave velocity, longitudinal wave velocity, density, Poisson’s ratio and Young’s modulus separately.

### 3.6. SHPB Experiment

#### 3.6.1. SHPB Experiment Layout

The SHPB dynamic compression experiment was carried out to determine the parameters of the Johnson–Cook constitutive equation. The ϕ 6 × 6 mm cylindrical specimens were prepared by SLM process, and the material composition and processing parameters are shown in Table 1 and Table 2. The test was carried out at room temperature regardless of the influence of temperature on the mechanical properties of the specimens. The dynamic compression experiment was carried out on a ϕ 14.5 mm SHPB, and the strain rate ranged from 1300 to 2400 s^−1^. Five sets of valid data were taken for each set of strain rates. The experimental principles and experimental details were not presented here.

Through the engineering to the true conversion showed as follows:

(4) $$\varepsilon_{true}=\ln\left(1+\varepsilon_{nom}\right)$$

(5) $$\sigma_{true}=\sigma_{nom}\left(1+\varepsilon_{nom}\right)$$

where $\varepsilon_{true}$, $\varepsilon_{nom}$ are true strain and engineering strain, respectively, and $\sigma_{true}$, $\sigma_{nom}$ are true stress and engineering stress, respectively.

**Figure 3:**
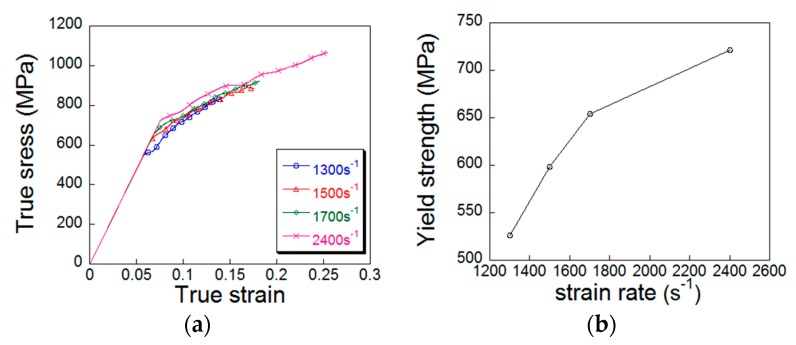
Plots of true stress–strain and yield strength vs. strain rate: (**a**) compressive stress–strain curve at room temperature; (**b**) yield strength vs. strain rate curve.

The true curve was plotted. Figure 3a shows the true curve converted by Equations (4) and (5) at room temperature and different strain rate loading conditions. Figure 3b shows the yield strength at different strain rates.

Figure 3b shows that the yield strength and flow stress value of the material gradually increases with the increase of the strain rate at room temperature.

#### 3.6.2. Determination of Parameters of Johnson–Cook

Based on the SHPB dynamic experimental data of AlSi10Mg specimens at room temperature, the material parameters of the Johnson–Cook equation were fitted using the least square method. The base strain rate was determined to be 1500 s^−1^. Subsequently, the parameters of Johnson–Cook constitutive model parameters of the specimen’s material obtained by the SHPB experiment are shown in Table 5.

## 4. Finite Element Simulation Analysis of SLM and UPT

**Figure 4:**
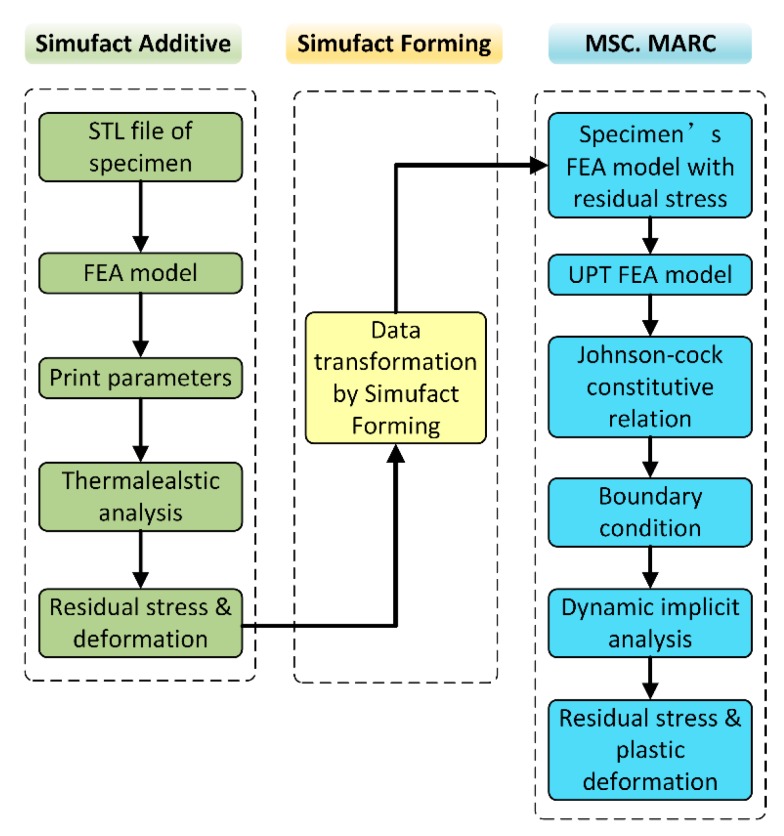
The diagram of SLM process–UPT simulation flowchart.

The finite element simulation analysis work in this paper was divided into two parts. Firstly, the three-dimensional thermomechanical coupling analysis of the laser selective melting process of the specimen was carried out by using a software Simufact Additive (3.0) to obtain the residual stress field distribution inside the specimen. Then the MSC Marc dynamics analysis software (2017) was used to simulate the UPT process of the specimen, and the variation of the residual stress field and the influence of different peening process parameters on the stress field distribution were investigated. The detailed simulation analysis procedure was shown in Figure 4.

### 4.1. The Simulation of SLM Fabrication Process of the Specimen

**Figure 5:**
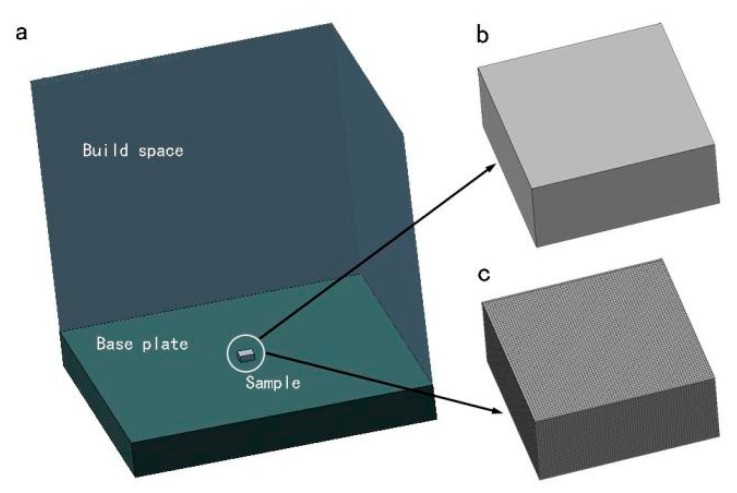
3D model of the specimen in Simufact Additive: (**a**) schematic of building setup; (**b**) unmeshed model; (**c**) meshed finite element (FEM) model.

AlSi10Mg specimens (12 × 12 × 4.5 mm) were prepared for UPT and residual stress measurement experiments by an AFS-M260 SLM machine. The SLM process parameters are shown in Table 2. Simufact Additive software was used to simulate the SLM build process. In the simulation, a 12 × 12 × 4.5 mm cuboid model was built and saved as a STL file first. Then the model was located on the substrate in a 260 × 260 × 350 mm build chamber, and the build orientation was determined as shown in Figure 5a. The specimen was meshed using 0.2 × 0.2 × 0.2 mm hexahedral elements. Figure 5c shows the meshed specimen model. Finally, the thermomechanical coupling simulation was carried out by Simufact Additive to obtain the residual stress distribution inside the specimen.

### 4.2. Simulation of UPT

#### 4.2.1. Finite Element Model of UPT

**Figure 6:**
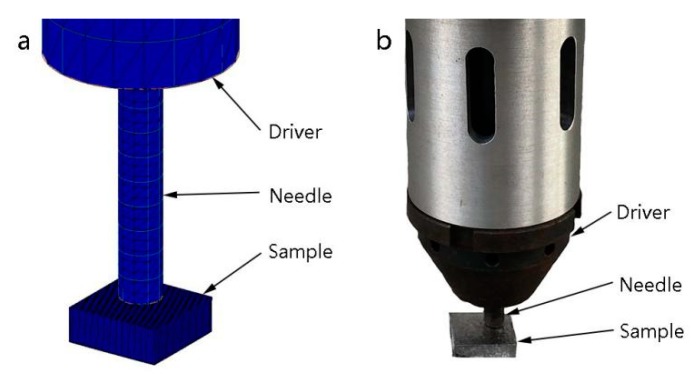
Schematic of UPT of the specimen: (**a**) 3D FEM model in MSC Marc; (**b**) Setup of the UPT experiment.

The finite element model of the SLM-fabricated specimen containing the residual stress and deformation distribution obtained in the previous simulation was processed by Simufact Forming software (15.0) as the initial finite element model of UPT analysis by MSC Marc software, and the amplitude transformer and peening needle model were established as well. Finally, a finite element model for the analysis of the ultrasonic peening process of SLM forming specimens was established. Figure 6 shows schematics of the ultrasonic peening 3D model and the experimental setup. The material of the amplitude transformer and the peening needle is Q235, and the mechanical properties of the material are shown in Table 6. In order to simplify the simulation, the material mechanical property of peening needle was regarded as rigid.

#### 4.2.2. Distance between the Peening Needle and the Specimen

**Figure 7:**
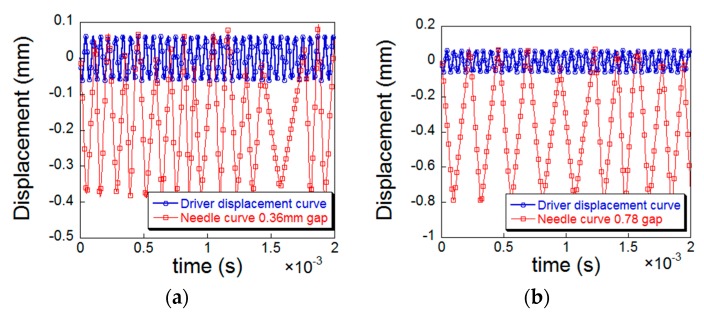

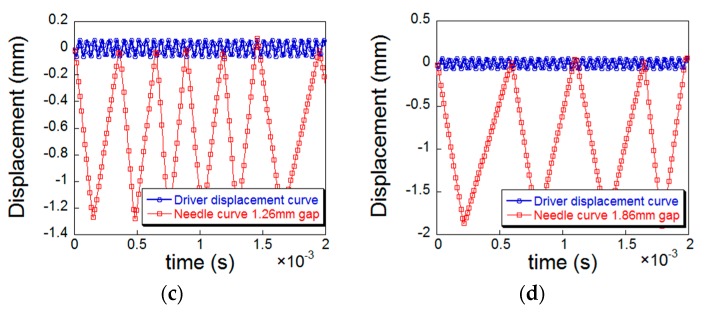
Displacement curve of the amplitude transformer and the needle with regard to different gap distance: (**a**) 0.36 mm; (**b**) 0.78 mm; (**c**) 1.26 mm; (d) 1.86 mm.

Statnikov et al. [6] found that the amplitude transformer can only deliver up to 25% of the energy to the peening needle. In order to make the simulation effect more realistic, it was necessary to adjust the distance between the peening needle and the specimen [26]. Four sets of data of 0.36, 0.78, 1.26 and 1.86 mm were selected as the distance between the amplitude transformer and the peening needle for simulation analysis, and separately extracted the displacement curves during the peening process to determine the peening needles frequency corresponding to different distance. Figure 7a–d are plots of the displacement of the amplitude transformer and peening needle over time during the simulation.

As shown in Figure 7, the frequency of the peening needle decreases with the increase of distance. When the distance is 0.78 mm, the frequency ratio of the peening needle to the amplitude transformer is about 25%, which is in accordance with the research results of Statnikov et al. [6] and Yin et al. [26], so the distance between the amplitude transformer and the peening needle was set as 0.78 mm in our simulations.

#### 4.2.3. Boundary Condition

In the simulation, the actual UPT process of the test specimen was simplified, and only a single peening process at the fixed position of the specimen was simulated. The single peening in the simulation corresponds to the full coverage of the 100% peening in the experiment [26]. As shown in Figure 7b, the time-displacement curve of the peening needle is extracted from the numerical simulation. It can be concluded that the time interval of one peening is about 2.4 × 10^−4^ s, and a complete peening process includes the needle impacting the top surface of the specimen after obtaining kinetic energy from the amplitude transformer and rebounding to contact with the amplitude transformer surface again. The peening duration time 4.8 × 10^−4^ s, 9.6 × 10^−4^ s, 1.68 × 10^−3^ s, 2.4 × 10^−3^ s were taken to compare the effect of the UPT duration time on the residual stress field.

**Figure 8:**
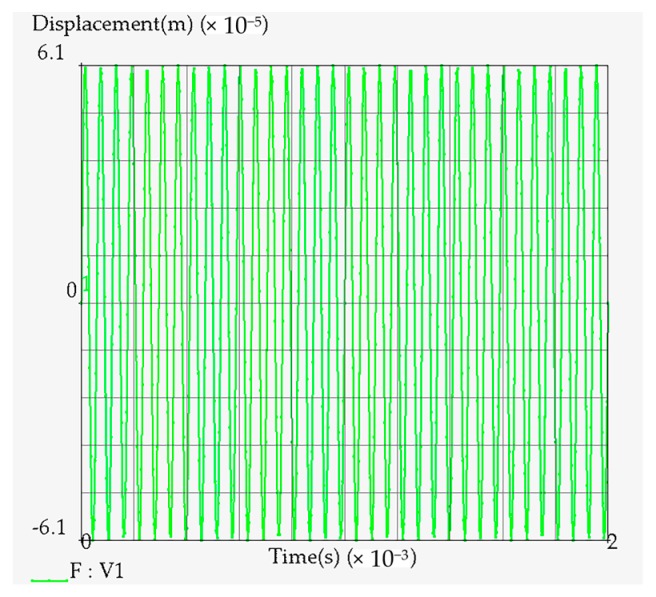
Displacement load applied on the amplitude transformer.

Figure 8 shows the displacement sinusoid applied to the amplitude transformer, time versus the change of displacement in MSC Marc software, the peening frequency input is a circular frequency, and its conversion relationship with the linear frequency is *w* = 2*πf*, *w* is the circular frequency, and *f* is the linear frequency. Then the sinusoidal displacement load of the amplitude transformer is *F* = 0.000061 × *sin*(106,760 × *V*1), where 0.000061 indicates that the amplitude of the amplitude transformer is 6.1 × 10^−5^ m, and 106,760 is the circular frequency of the amplitude transformer vibration.

The bottom surface of the test piece was completely constrained by *x* = *y* = *z* = 0, the degree of freedom of the peening needle in the *x*-, *y*-axis direction was zero *x* = *y* = 0, and the specimens were peened along the *z*-axis direction.

## 5. Results and Discussions

### 5.1. The Comparison of Measured and Simulated Residual Stress

The time duration of a single peening in the simulation can be extracted from the time-displacement history curve. According to Section 4.2.3, the single peening in the simulation can correspond to a full coverage of 100% peening in the experiment. In the experiment, the full coverage peening time was measured as 5 s, which is equivalent to a single peening time of 2.4 × 10^−4^ s in the simulation. The total peening time of this test is 50 s, which is equivalent to a continuous peening at the fixed position for 2.4 × 10^−3^ s in the simulation.

**Figure 9:**
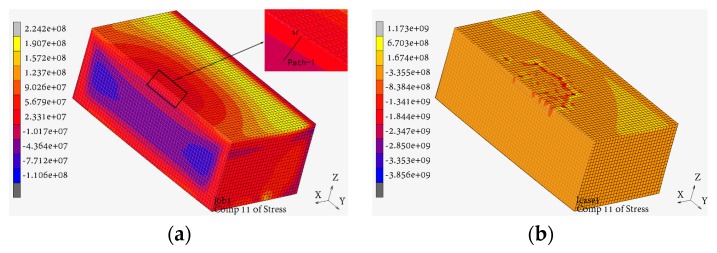
Residual stress (Pa) contour plots by simulation: (**a**) Residual stress contour plot of the specimen as-fabricated by SLM machine; (**b**) Residual stress contour plot of the specimen after UPT.

**Figure 10:**
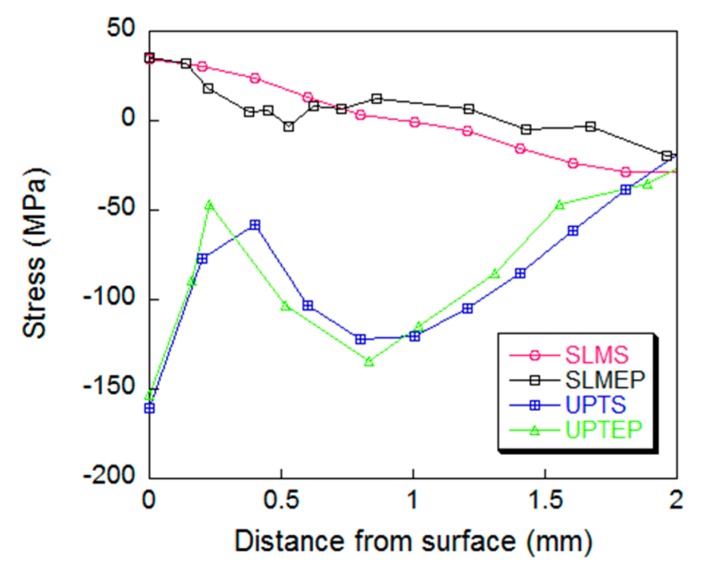
Comparison of residual stress of the specimen by simulation and measurement, curves SLMS and SLMEP represent residual stresses of specimen as-fabricated by SLM by simulation and measurement respectively, curves UPTS and UPTEP represent residual stresses of specimen after UPT by simulation and measurement respectively.

The parameters of the Johnson–Cook constitutive model were determined by the previous SHPB test, and the model was used to describe the material mechanical properties of the specimen in the simulation, considering the influence of UPT effect on the mechanical properties of the material. The residual stress distributions in the specimen obtained by the simulation before and after UPT were shown in Figure 9. Half of the specimen was taken to show the residual stress cloud diagram of the middle section. The comparisons of residual stresses measured by the experiment and obtained in the simulation were shown in Figure 10.

As shown in Figure 9, the residual stress field is more uniform after UPT process compared with an as-fabricated specimen.

Figure 10 indicates that the UPT process can effectively convert the residual tensile stress on the surface of the specimen into compressive stress. The maximum conversion difference is 205 MPa, and the effective action depth is 1.5–2 mm. Comparing the simulation results with the experimental results under the same conditions, the difference of residual stress on the surface of the specimen after UPT is 6.7 MPa, and the maximum deviation of the stress curve obtained by simulation and measurement is 26 MPa. It can also be seen that the simulated residual stress curve is offset to the right by a small distance from the measured, which may be attributed to the peeling measurement error. In general, the simulation results and the experimental measurement results of the specimens before and after the UPT are in good agreement as to the values and trends, which also verify the reliability of the SLM process simulation and the UPT process simulation of the specimen.

### 5.2. Influence of Peening Time on Residual Stress Field

The influence of peening time on the residual stress of the specimen was analyzed by numerical simulation method. The peening time duration was 4.8 × 10^−4^ s, 9.6 × 10^−4^ s, 1.68 × 10^−3^ s, 2.4 × 10^−3^ s respectively for comparative analysis. The surface residual stresses along the thickness direction at the center of specimen are shown in Figure 10 with the depth up to 2.3 mm.

**Figure 11:**
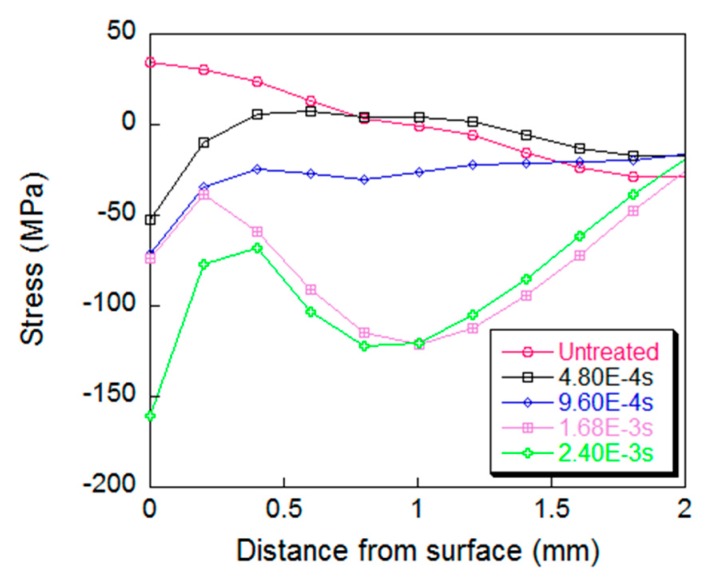
Plots of residual stress varied with the peening time duration.

It can be seen from Figure 11 that both the depth of action and the change of residual stress increase over the UPT time. When the UPT time is short, the residual stress values in the depth range of 1.5 mm from the surface change obviously, and the effect of UPT in the thickness direction decreases with the extension of the UPT time. However, as the UPT time proceeds, the trend changes. In the range of 0.5 mm depth from the surface, the effect of UPT decreases, and then the effect of the UPT on the residual stresses increases with the depth, reaching an extreme value at a depth of 1 mm, and then it decreases with increase of depth, and the maximum action depth can reach 2 mm. The residual stress value beyond the 2-mm depth from the surface does not change with the extension of the UPT time. The reason may be that the surface layer of the specimen generates plastic deformation during the UPT process, resulting in a work hardening effect, and the specific reasons need further investigations.

### 5.3. Influence of Peening Amplitude

**Figure 12:**
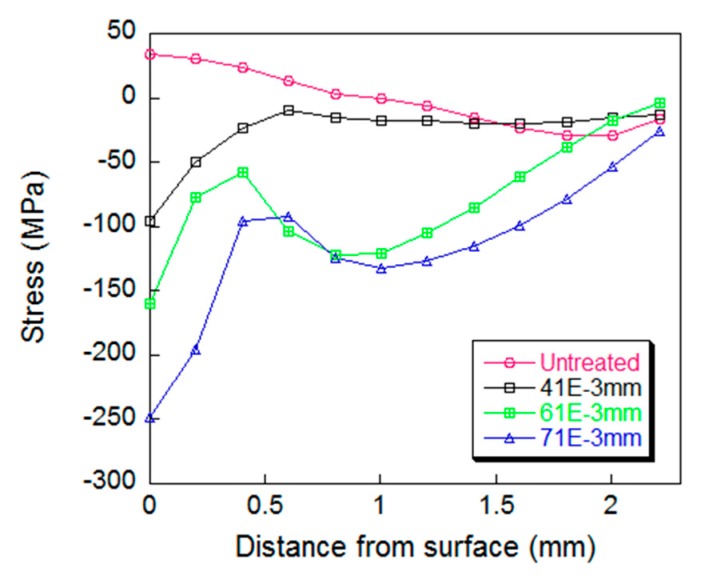
Plots of residual stress with regards to different amplitude.

The amplitude was the main process parameter of the UPT equipment. Under the conditions of constant frequency, peening time, distance between the peening needle and the specimen, the UPT process simulations of the specimen were carried out by varying amplitude to 41, 61 and 71 μm. Then, the residual stress curves varied with the depth were plotted as shown in Figure 12.

**Figure 13:**
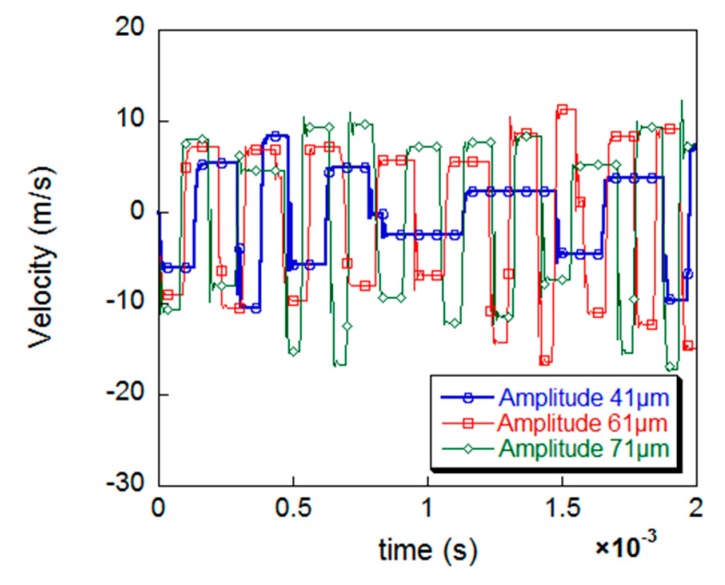
Plots of time-velocity curve at different amplitude.

Figure 12 shows the action depth of the UPT and the change of the residual stress values increase with the increase of the peening amplitude. When the amplitude reaches 71 μm, compression stress of up to 250 MPa was generated in the surface of the specimen after UPT, and the residual stress changes significantly compared to the case of 41 μm amplitude. The time-velocity curve of the peening needle under different amplitudes is shown in Figure 13. It can be seen that as the amplitude increases, the initial velocity of the peening needle and the velocity at the moment of impacting the specimen increase, which means that more dynamic energy of the needle will be transmitted to the specimen. That is why the effect of UPT increases with amplitude.

### 5.4. Influence of Peening Frequency

**Figure 14:**
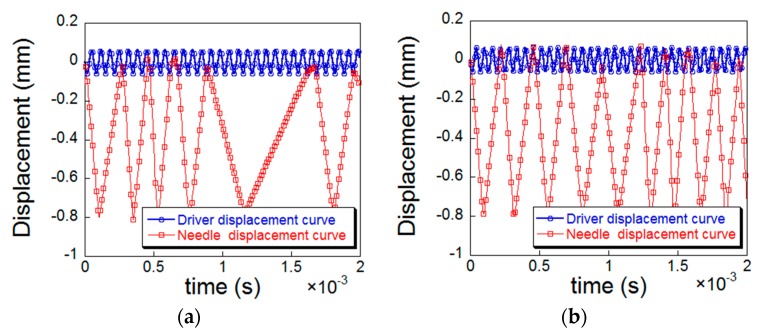

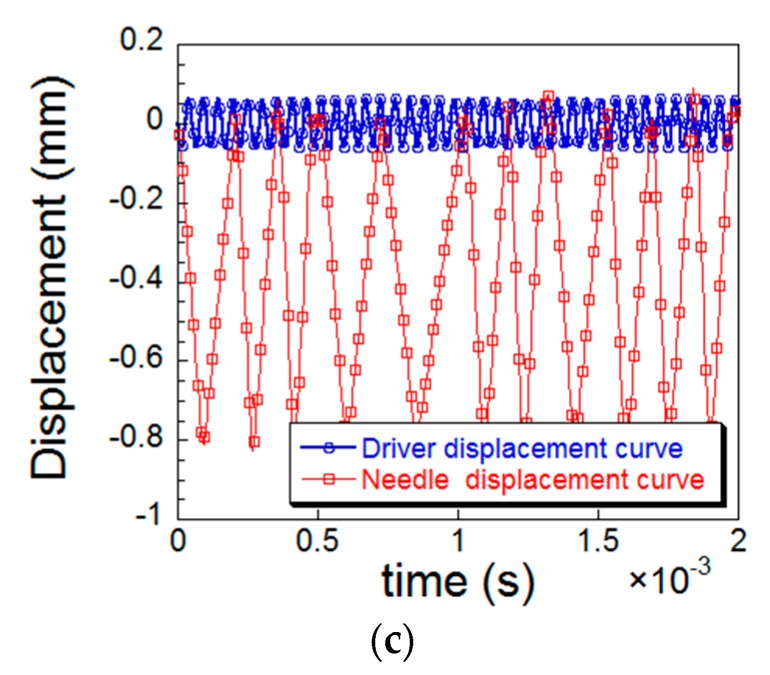
Plots of displacement vs. time at different frequency: (**a**) 15 KHz; (**b**) 17 KHz; (**c**) 19KHz.

**Figure 15:**
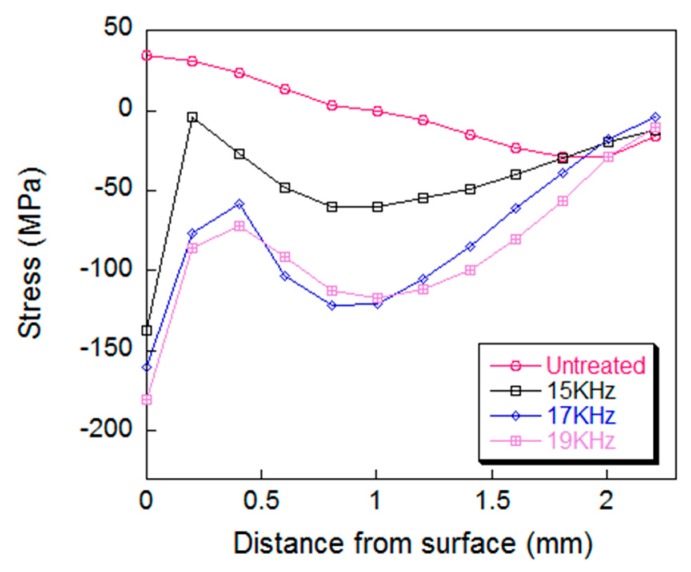
Plots of residual stress curve with regards to frequency.

The peening frequency was another main process parameter of the UPT equipment. It can be seen from Figure 14 a–c that as the frequency of the amplitude transformer increases, the frequency of the peening needle also increases, which means that the specimen is impacted more times in the same peening time duration. The UPT process simulations are conducted with respect to common frequencies of 15 KHz, 17 KHz, and 19 KHz. The residual stress versus depth curves of the specimen after UPT are shown in Figure 15.

It can be seen in Figure 15 that as the peening frequency increases, the effect of UPT on the residual stress increases, but there is no significant influence on the depth of action.

## 6. Conclusions

In this paper, the SLM building process of AlSi10Mg specimens, the UPT process of the specimen and the effect of UPT on the residual stress distribution were studied by means of experiment and FEM simulation. The UPT process can significantly modify the residual stresses distribution in the laser additively manufactured specimens, the tensile stresses (the maximum value 50 MPa) can be changed into compressive stresses (the minimum value −150 MPa) after UPT. An experimental study strategy and setup was presented first. A FEM method for simulating the UPT process of the specimens was proposed. Simufact Additive was used to simulate the SLM building process and predict the residual stress field. The specimen’s model file containing residual stress was processed using Simufact Forming, and then it was applied as the initial FEA model for UPT simulation in MSC Marc. Based on the initial prestressed specimen model, a dynamic finite element model of UPT established in MSC Marc was used to analyze the influence of UPT on residual stress. The material mechanical property data and constitutive model parameters required in the simulation analysis were obtained through a series of experiments. The Young’s modulus and Poisson’s ratio of the specimens were measured by ultrasonic detector measurement method. The parameters of Johnson–Cook constitutive model were determined by SHPB experiments, and the model was used to define the material properties of the specimens in the MSC Marc software. The residual stress obtained by the simulation agrees well with the experimental measurements, and the simulation model and simulation strategy are proved to be reliable. On this basis, the influence of parameters such as time, amplitude and frequency of ultrasonic peening on residual stress was discussed by numerical simulation method. Increasing the peening time, amplitude and frequency can increase the effect of ultrasonic shock on residual stress. The influence of time and peening amplitude on the residual stress is greater, and the maximum depth of action could reach 2 mm.

## Figures and Tables

**Table 1:** The chemical composition of AlSi10Mg (wt %).

Materials	Al	Si	Mg	Fe	Cu	Mn	Zn	Other
AlSi10Mg	Balance	9.0–10.0	0.40–0.60	≤2.0	≤0.60	≤0.35	-	≤0.25

**Table 2:** SLM process parameters.

Materials	Layer Thickness	Laser Power	Speed	Spot Diameter	Hatching Space	Scanning Width
AlSi10Mg	0.03 mm	200 W	1.8 m/s	0.07 mm	0.06 mm	4 mm

**Table 3:** Parameters of UPT.

Frequency	Power	Amplitude	Current	Impact Time
17 KHz	1000 W	61 μm	2.2 A	60 s

**Table 4:** Mechanical property parameters of the specimen fabricated by a SLM machine.

Material	Density	Young’s Modulus	Poisson’s Ratio
AlSi10Mg	2.68 g/cm^3^	81.97 GPa	0.3

**Table 5:** Parameter values of Johnson–Cook model obtained from SHPB experiments.

Strain Rate	*A*	*B*	*n*	*C*	*m*
1500 s^−1^	598 MPa	1509 MPa	0.928	0.0375	1

**Table 6:** Mechanical parameters of material.

Materials	Density	Young’s Modulus	Passion’s Ratio
Q235	7800 Kg/m^3^	210 GPa	0.3
